# Impaired Dynamic Cerebral Autoregulation in Cerebral Venous Thrombosis

**DOI:** 10.3389/fneur.2020.570306

**Published:** 2020-11-09

**Authors:** Jie Chen, Jia Liu, Kehui Dong, Yilong Wang, Xingquan Zhao, Yongjun Wang, Xiping Gong

**Affiliations:** ^1^Vascular Neurology, Department of Neurology, Beijing Tiantan Hospital, Capital Medical University, Beijing, China; ^2^Shenzhen Institutes of Advanced Technology, Chinese Academy of Sciences, Shenzhen, China

**Keywords:** homeostasis, cerebral venous thrombosis (CVT), transcranial doppler (TCD), cerebral autoregulation (CA), transfer function

## Abstract

**Background:** Cerebral autoregulation is crucial in traumatic brain injury, which might be used for determining the optimal intracranial pressure. Cerebral venous thrombosis (CVT) is a cerebral vascular disease with features of high intracranial pressure. However, the autoregulatory mechanism of CVT remains unknown. We aimed to investigate the capacity of cerebral autoregulation in patients with CVT.

**Methods:** This study consecutively enrolled 23 patients with CVT and 16 controls from December 2018 to May 2019. Cerebral autoregulation was assessed by transfer function analysis (rate of recovery/phase/gain) using the spontaneous oscillations of the cerebral blood flow velocity and arterial blood pressure.

**Results:** In total, 76 middle cerebral arteries (MCAs) were investigated, including 44 MCAs in patients with CVT and 32 normal ones. The phase shift estimated in patients with CVT was significantly different from that of the controls (37.37 ± 36.53 vs. 54.00 ± 26.78, p = 0.03). The rate of recovery and gain in patients with CVT were lower than those in controls but without statistical significance.

**Conclusion:** To our knowledge, this is the first time that a study has indicated that patients with CVT were more likely to have impaired cerebral autoregulation. Hence, cautious blood pressure control is required in such patients to prevent hyper- or hypoperfusion.

## Introduction

Cerebral venous thrombosis (CVT) is a relatively rare cerebrovascular disease, usually affecting young individuals (1). CVT has highly variable clinical presentations, including headaches, visual impairment, focal neurological deficits, seizures, and encephalopathy. Hence, the diagnosis and management of CVT might be difficult. Although the clinical presentations vary, the pathogenesis of CVT is partly caused by elevated intracranial pressure, obstruction of the venous blood drainage, and decreased cerebrospinal fluid absorption (2).

Many studies have shown that intracranial hypertension may impair the mechanism of cerebral autoregulation in patients with subarachnoid hemorrhage and traumatic brain injury (3–7). A previous meta-analysis that reviewed 16 relevant studies stated that patients with intracranial hypertension had increased risk of impaired autoregulation (8). Although autoregulation is a key mechanism to maintain the cerebral blood flow relatively constant, despite variations in blood pressures, impaired cerebral autoregulation may lead to passive changes in cerebral blood flow in response to the fluctuations of blood pressure and increase the risk of brain hyperperfusion or hypoperfusion (9).

It remains unknown whether CVT might impair the mechanism of cerebral autoregulation. Hence, we conducted this study to investigate the cerebral autoregulation in patients with CVT.

## Materials and Methods

### Patients and Controls

In this study, 23 patients with CVT [12 male, 11 female; age 34 ± 16 years (mean ± SD)] were consecutively recruited from December 2018 to May 2019. In addition, 16 age-matched healthy individuals (9 male, 7 female; age, 40 ± 11 years) were recruited as controls. All control subjects underwent a transcranial Doppler (TCD) examination to exclude carotid artery or intracranial stenosis (10).

### Autoregulation Measurements

Cerebral blood flow velocity (CBFV) was measured in the bilateral middle cerebral arteries (MCAs) using a TCD (DWL, Germany). Continuous beat-to-beat arterial blood pressure (ABP) was recorded by a servo-controlled finger plethysmograph (AD instruments, Australia). After the baseline value stabilized, the data of the CBFV and ABP were recorded at a sample rate of 100 Hz for at least 5 min with the participants breathing spontaneously in supine position.

### Transfer Function Analysis

Cerebral autoregulation was evaluated using transfer function analysis (11, 12). The Welch's method was used to estimate the auro-spectrum of the ABP and the oss-spectrum of the ABP and CBFV as follows:

(1) $$H(f)=\;S_{xy}/S_{xx}$$

The phase and gain were calculated as follows:

(2) $$\varphi (f)={\text{tan}}^{-1}\left[H_{I}(f)/H_{R}(f)\right],$$

(3) $$\left|H(f)\right|=\;\sqrt{{\left[H_{I}(f)\right]}^{2}+{\left[H_{R}(f)\right]}^{2}}$$

The φ(*f*) and |*H*(*f*)| denote the phase response and gain of the transfer function, respectively.

The coherence function, *C*_*xy*_, which indicates how well the CBFV corresponds to ABP in frequency, was computed as:

(4) $$C_{xy}=\;\frac{{\left|\;S_{xy}\right|}^{2}}{S_{xx}S_{yy}}$$

In frequency domain, we estimated phase shift, gain, and coherence function within 0.06–0.12 Hz to evaluate cerebral autoregulation (13).

In the time domain, the step response of the CBFV was estimated by the convolution between the impulse and the step change of the ABP, for showing the recovery of the CBFV after the ABP changed stepwise. The rate of recovery (RoRc) of the CBFV (the first 3 s of the step response) was calculated for quantification of the speed of recovery as follows (14):

(5) $$\begin{array}{r} \text{RoRc}=\frac{\Delta \text{CBFV}}{\Delta \text{t}}\times 100\% \end{array}$$

### Statistical Analysis

SPSS 25.0 (SPSS Inc., USA) was used for the statistical analysis. The Student's *t*-test was used to compare the continuous variables across groups, and Pearson χ^2^ was used to compare categorical variables between groups. A *p* < 0.05 was considered statistically significant.

## Results

There was no significant difference between cases and controls in age, sex, and history of hypertension (Table 1). Among the 23 recruited patients, headache was the most frequent symptom, followed by focal neurological deficits and seizures. A total of six patients were in pregnancy and puerperium; other risk factors included dehydration, oral contraceptives, inflammatory diseases, hematological condition, and dural fistulae. The mean time from onset to TCD examination was 20.50 ± 27.94 days, range from 1 to 120 days. The clinical characteristics are summarized in Table 1.

**Table 1:** Clinical characteristics of patients with cerebral venous thrombosis and controls.

	**CVT patients (*n* = 23)**	**Controls (*n* = 16)**	***p***
Age, years	34.26 ± 16.31	40.31 ± 10.75	0.202
Male, *n* (%)	12 (52.2)	9 (56.25)	0.802
Hypertension, *n* (%)	8 (34.78)	4 (25.00)	0.515
**Clinical manifestation**, ***n*** **(%)**			
Headache	21 (91.3%)		
Focal neurological deficits	10 (43.5)		
Seizures	9 (39.1%)		
Visual impairment	6 (26.1%)		
Consciousness disturbances	4 (17.4%)		
**Risk factors**			
Pregnancy and puerperium	6 (26.1%)		
Dehydration	3 (13.0%)		
Oral contraceptives	2 (8.7%)		
Inflammatory diseases	2 (8.7%)		
Hematological condition	1 (4.3%)		
Dural fistulae	1 (4.3%)		

*CVT, cerebral venous thrombosis*.

All participants underwent examination of bilateral MCAs, except for two patients with one inadequate temporal window. Thus, 76 MCAs were investigated, including 44 MCAs in patients with CVT and 32 in controls.

**Figure 1:**
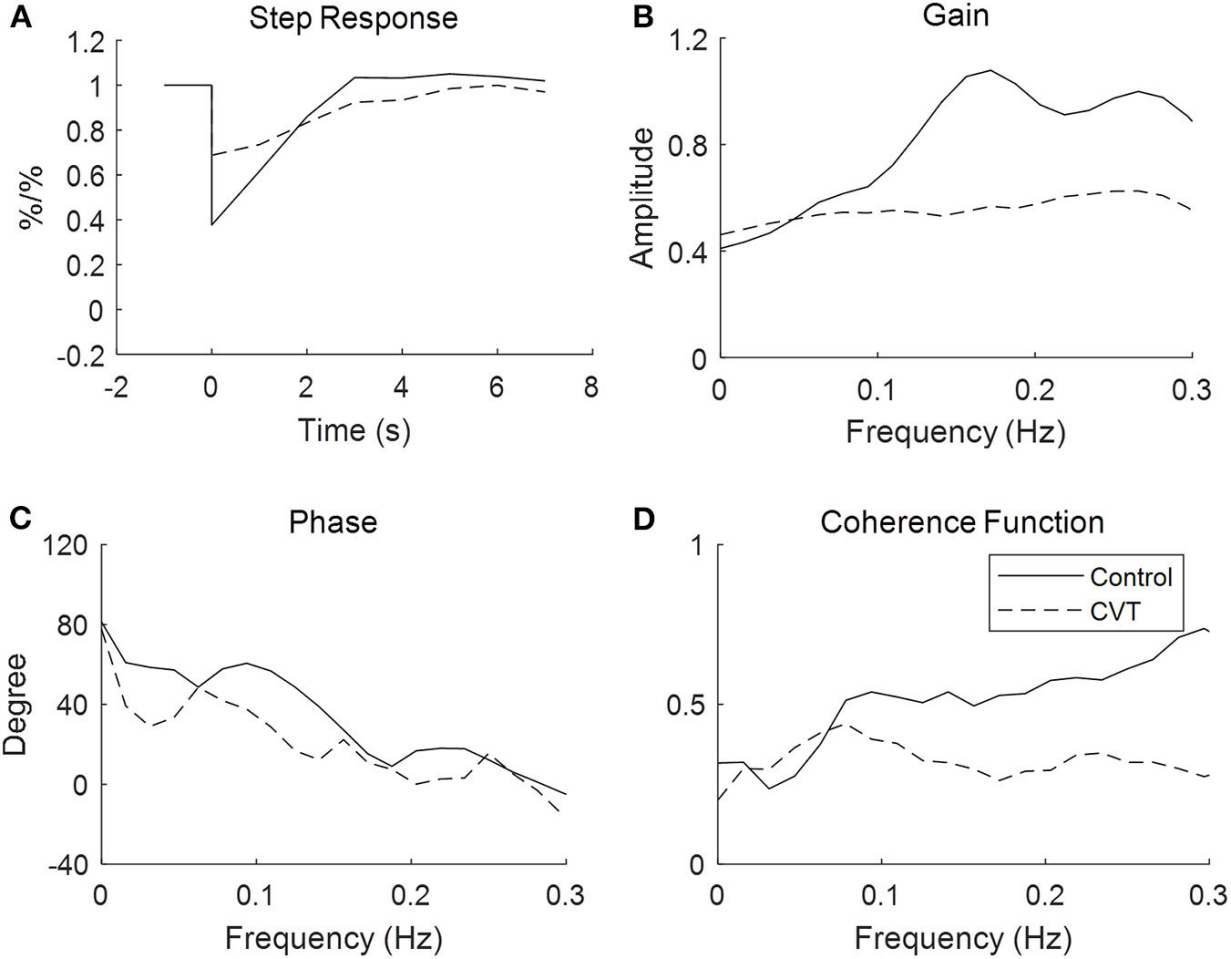
Transfer function analysis of cerebral autoregulation in controls and patients with cerebral venous thrombosis. The figures are the averaged step response, gain, phase, and coherence function for the controls and patients, respectively, with cerebral venous sinus thrombosis derived from the transfer function.

In the time domain, the averaged step response of the controls reached the baseline level within 3 s, while it took a longer time (about 6 s) for patients with CVT. This indicated that if the ABP dropped in a step-wise manner, the CBFV in patients with CVT recovered more slowly that in the controls (Figure 1). In addition, the RoRc of patients with CVT was significantly lower than that of the controls (29.74 ± 59.55 vs. 37.95 ± 22.55, *p* = 0.01, Table 2).

**Table 2:** Results of rate of recovery, magnitude, phase shift, and coherence in controls and patients with cerebral venous thrombosis (CVT).

	**Controls (*n* = 32)**	**CVT patients (*n* = 44)**
RoRc (%/s)	37.95 ± 22.55	29.74 ± 59.55^*^
Gain (ampl.)	0.62 ± 0.33	0.54 ± 0.35
Phase (degree)	54.49 ± 26.44	37.63 ± 33.94^*^
Coherence	0.44 ± 0.17	0.39 ± 0.20

* *p < 0.05, compared with normal controls*.

*CVT, cerebral venous thrombosis; RoRc, rate of recovery*.

With respect to the transfer function analysis in the frequency domain, the phase shift at 0.06–0.12 Hz was significantly lower in the patients with CVT than in the controls (37.63 ± 33.94 vs. 54.49 ± 26.44, p = 0.23, Figure 1, Table 2). The gain was lower in patients with CVT than in the controls (0.54 ± 0.35 vs. 0.62 ± 0.33); however, this difference was not statistically significant (*p* = 0.08, Table 2).

Magnetic resonance venogram revealed that the most frequent location of CVT was the lateral sinus (74.0%), followed by the sagittal sinus (65.2%), sigmoid sinus (52.2%), straight sinus (34.8%), jugular veins (17.4%), and the cerebral deep venous system (8.7%). There were eight patients with involvement of a unilateral sinus and 15 patients with bilateral sinus involvement. All the autoregulatory parameters analyzed showed no statistic differences between the 36 affected hemispheres (except in two patients with inadequate windows) and the eight non-affected hemispheres (phase shift: 37.79 ± 34.28 vs. 23.00 ± 28.36, *p* = 0.21).

Among all the included patients with CVT, 14 patients (60.1%) had brain parenchymal lesions, including cerebral ischemia (39.1%), intracranial hemorrhage (43.5%), and subarachnoid hemorrhage (4.3%). Thus, 21 hemispheres had focal lesions (except two with an inadequate window), and 23 hemispheres had none. No statistical differences were found for the phase shift and RoRc between the hemispheres with and without lesions (41.19 ± 28.53 vs. 29.54 ± 37.20, *p* = 0.07; 22.14 ± 58.05 vs. 27.36 ± 60.73, *p* = 0.53, respectively). However, there were statistical differences in the phase shift and RoRc between the hemispheres in CVT patients without lesions and in the controls (29.54 ± 37.20 vs. 54.49 ± 26.44, *p* = 0.01; 27.36 ± 60.73 vs. 37.95 ± 22.55, *p* = 0.006, respectively).

## Discussion

In this study, the phase shift of the transfer function analysis showed a significant reduction in patients with CVT, which indicated that cerebral autoregulation was impaired in such patients.

In patients with CVT, obstruction of the venous blood drainage and decreased cerebrospinal fluid absorption cause elevated intracranial pressure. This can subsequently lead to venous cerebral infarction and hemorrhage, which then damages the cerebral arterioles and capillaries (2). The cerebral arterioles and capillaries form the effectors of cerebral autoregulation through complex myogenic, metabolic, and neurogenic mechanisms (9). Hence, this might be the reason for impaired cerebral autoregulation in patients with CVT.

In our study, brain parenchymal lesions, including cerebral ischemia, hemorrhage, and subarachnoid hemorrhage, were found in 44.7% of the hemispheres, and these lesions may impair the autoregulative capacity (15–17). However, our results showed that the cerebral autoregulation deteriorated even in the hemispheres without lesions, and there were no differences between the hemispheres with and without lesions. Furthermore, bilateral impairment of autoregulation was noted, which might also cause impaired cerebral autoregulation in patients with CVT.

In the present study, the autoregulatory capacity was significantly impaired in patients with CVT. Therefore, the blood pressure of patients with CVT should be managed carefully, as cerebral hemodynamics might become vulnerable to the fluctuations of ABP. The elevations in ABP might result in hyperperfusion, which may in turn elevate the intracranial pressure, which conversely worsens the autoregulatory capacity and thus creates a vicious circle. In contrast, a fall in ABP might induce hypoperfusion and cerebral ischemia. Therefore, assessment of cerebral hemodynamics should be considered in patients with CVT, especially for the guidance of hypertensive therapy. Many studies have revealed that impaired cerebral autoregulation is an independent impactor of unfavorable neurological outcomes in patients with stroke, subarachnoid hemorrhage, traumatic brain injury, and carotid or intracranial stenosis (15–18). Recent studies have shown that in patients with traumatic brain injury, the optimal intracranial pressure could be determined by assessing the cerebral autoregulation, and this could be used to adjust the intracranial pressure for favorable outcomes (19). In the future, we hope that new models will be developed for determining the optimal blood pressure by analyzing autoregulation, which will allow better blood pressure management in patients with CVT.

This study has several limitations. First, it is a monocenter pilot investigation with a relatively small sample size. Although the baseline characteristics were similar between patients and controls, imbalance cannot be ruled out. Second, there is time delay between onset of clinical symptoms and TCD examination, and some patients were not in the acute phase of CVT, which may influence the measuring accuracy of cerebral autoregulation. Further studies for dynamic assessment of autoregulatory function at different stages of CVT patients are needed.

In conclusion, our study suggests that impaired cerebral autoregulation occurs in patients with CVT. Further investigation is required to determine whether autoregulation may be of potential clinical value in the management of patients with CVT.

## Data Availability Statement

The raw data supporting the conclusions of this article will be made available by the authors, without undue reservation.

## Ethics Statement

The studies involving human participants were reviewed and approved by Beijing Tiantan Hospital Research Ethics Committee. The patients/participants provided their written informed consent to participate in this study.

## Author Contributions

XG, YoW, XZ, and YiW designed the study. JC, XG, and KD gathered the data. JL and JC conducted the data analysis. JC and XG drafted the manuscript. All authors contributed to the article and approved the submitted version.

## Conflict of Interest

The authors declare that the research was conducted in the absence of any commercial or financial relationships that could be construed as a potential conflict of interest.
